# The Host Specificities of Baculovirus *per os* Infectivity Factors

**DOI:** 10.1371/journal.pone.0159862

**Published:** 2016-07-25

**Authors:** Jingjiao Song, Xi Wang, Dianhai Hou, Huachao Huang, Xijia Liu, Fei Deng, Hualin Wang, Basil M. Arif, Zhihong Hu, Manli Wang

**Affiliations:** 1 State Key Laboratory of Virology, Wuhan Institute of Virology, Chinese Academy of Sciences, Wuhan 430071, P. R. China; 2 Experimental Medicine Center, Tongji Hospital, Tongji Medical College, Huazhong University of Science and Technology, Wuhan 430030, P. R. China; 3 Laboratory for Molecular Virology, Great Lakes Forestry Centre, Sault Ste. Marie, Ontario, Canada; Wuhan University, CHINA

## Abstract

Baculoviruses are insect-specific pathogens with a generally narrow host ranges. Successful primary infection is initiated by the proper interaction of at least 8 conserved *per os* infectivity factors (PIFs) with the host’s midgut cells, a process that remains largely a mystery. In this study, we investigated the host specificities of the four core components of the PIF complex, P74, PIF1, PIF2 and PIF3 by using Helicoverpa armigera nucleopolyhedrovirus (HearNPV) backbone. The four *pif*s of HearNPV were replaced by their counterparts from a group I Autographa californica multiple nucleopolyhedrovirus (AcMNPV) or a group II Spodoptera litura nucleopolyhedrovirus (SpltNPV). Transfection and infection assays showed that all the recombinant viruses were able to produce infectious budded viruses (BVs) and were lethal to *H*. *armigera* larvae via intrahaemocoelic injection. However, feeding experiments using very high concentration of occlusion bodies demonstrated that all the recombinant viruses completely lost oral infectivity except SpltNPV *pif3* substituted *pif*3-null HearNPV (vHaBacΔ*pif*3-Sp*pif*3-*ph*). Furthermore, bioassay result showed that the median lethal concentration (LC_50_) value of vHaBacΔ*pif*3-Sp*pif*3-*ph* was 23-fold higher than that of the control virus vHaBacΔ*pif*3-Ha*pif*3-*ph*, indicating that SpltNPV *pif*3 can only partially substitute the function of HearNPV *pif*3. These results suggested that most of PIFs tested have strict host specificities, which may account, at least in part, for the limited host ranges of baculoviruses.

## Introduction

Baculoviruses are insect-specific large DNA viruses, which have important applications in the areas of insect pesticides, protein expression and gene therapy [1–3]. The *Baculoviridae* family is constituted of four genera: *Alpha-*, *Beta-*, *Gamma-* and *Delta-baculovirus*. A typical life cycle of apha- and betabaculoviruses produces two morphologically distinct virions: the budded virus (BV) and the occlusion-derived virus (ODV) [4]. ODVs enter the epithelial cells of insect midgut through direct membrane fusion to initiate primary infection, while BVs are transmitted from cell to cell and cause systemic infection [5, 6].

The successful initiation of infection in the midgut epithelium by the ODVs is largely dependent on a number of virus encoded proteins termed *per os* infectivity factors (PIFs). So far, eight *pif* genes have been identified to be conserved in all sequenced baculoviruses, including *p74* [7, 8], *pif1* [9], *pif2* [10, 11], *pif3* [12, 13], *pif4* [14, 15], *pif5* [16, 17], *pif6* [18] and pif7 [19]. Absence of any *pif* gene will result in a profound impairment or complete loss of oral infectivity. All PIFs except PIF7 are conserved in baculoviruses, while PIF7 is conserved only in lepidopteran baculoviruses (alpha- and betabaculoviruses). Interestingly, homologues of a few PIFs are also found in nudiviruses, salivary gland hypertrophy viruses (SGHVs) and the white spot syndrome virus (WSSV), implying an evolutionarily conserved ancient entry mechanism of invertebrate viruses [20].

Among the 8 PIFs, P74, PIF1-3, 5 are all ODV envelope-specific proteins [9, 15, 16, 21, 22], whereas PIF4 and PIF6 were detected in the envelope fractions of both budded virus (BV) and ODV [14]. PIF7 was suggested to be localized in the envelope fractions of ODV by a mass spectrometry study [23]. In Helicoverpa amigera nucleopolyhedrovirus (HearNPV), all the PIFs located in ODV envelope [23]. Recent studies have showed that P74 and PIF1-4 form a protein complex on the surface of ODV. PIF1-3 appear to be the core components, while P74 may be loosely associated with the complex [24, 25]. The PIF complex is likely to play an important role in virus entry into midgut epithelial cells of susceptible insect larvae [24]. Previous studies showed that P74, PIF1 and PIF2 are involved in the binding of ODV onto midgut cells [12, 26]. In contrast, PIF3 may not participate in virus binding process, instead, it is speculated to mediate nucleocapsid translocation along microvilli [12, 27].

Since PIFs are responsible for oral infection, it is reasonable to suggest their involvement in the host range of baculovirus. Unfortunately, studies of the specificity of PIFs are rather scanty. Wu *et al*. [28] generated a recombinant Autographa californica multiple nucleopolyhedrovirus (AcMNPV) with its *p74* gene replaced by that of Spodoptera litura nucleopolyhedrovirus (SpltNPV), but they did not test the oral infectivity of this recombinant virus, which should have been the logical experiment to do. Harrison *et al*. [16] found that replacement of AcMNPV *odv-e56* (*pif5*) gene with its counterpart from a closely-related Rachiplusia ou multiple nucleopolyhedrovirus (RoMNPV) did not increase virulence against larvae that are more susceptible to RoMNPV than to AcMNPV.

In this study, we tested whether P74, PIF1, PIF2 and PIF3 of HearNPV could be functionally substituted by their homologues from other baculoviruses. HearNPV is a group II alphabaculovirus specific to certain species of *Heliothis* [29]. The *pif*s of AcMNPV or SpltNPV were amplified and inserted into the *pif*s-deleted HearNPV bacmid. AcMNPV is a group I alphabaculovirus with wide host range of 39 lepidopteran species [30], while SpltNPV is a group II alphabaculovirus that infects a single host [31]. All the recombinant viruses produce infectious BVs, which are lethal to *H*. *armigera* lavae via intrahaemocoelic injection. However, bioassay results demonstrated that most recombinant viruses lost their oral infectivity completely, except SpltNPV *pif3* substituted *pif*3-null HearNPV (vHaBacΔ*pif*3-Sp*pif*3-*ph*) which retained only partial oral infectivity. These results revealed the involvement of PIF proteins in host specificities of baculovirus.

## Material and Methods

### Insect cells, insects and viruses

The *Helicoverpa zea* ovarian cell line HzAM1 [32] was maintained at 28°C in Grace’s medium (Gibco-BRL) supplemented with 10% fetal bovine serum. *H*. *armigera* larvae were reared on an artificial diet at 27°C. An infectious HearNPV bacmid HaBacHZ8, as well as *pif*s-deletion bacmids HaBacΔ*pif*s-*ph*, Ha*pif*s-repaired viruses vHaBacΔ*pifs*-Ha*pif*s*R*-*ph* and control virus vHaBac-*egfp*-*ph* were constructed previously in our laboratory (*ph* stands for *polyhedrin* gene) [22, 33]. HearNPVG4 strain, AcMNPV and SpltNPV were maintained as laboratory stocks.

### Construction of *pifs-*pseudotyped HearNPV bacmids

To construct bacmids with substituted *pifs*, the coding sequence along with the putative promoter region of *p74*, *pif1*, *pif2* or *pif3* were amplified from genomic DNA of AcMNPV and SpltNPV by specific primers listed in Table 1. The PCR products were cloned into pGEM-T easy vector (Promega) for sequencing. Then, these *pif* genes were inserted into the indicated restriction enzyme sites (Table 1) of the transfer vector pFB-DUAL-*ph* and further transposed into the respective *pif*-deletion HearNPV bacmids according to the Bac-to-Bac manual (Invitrogen). The resulting bacmids were identified by PCR analyses and designated as HaBacΔ*p74*-Ac*p74*-*ph*, HaBacΔ*pif1*-Ac*pif1*-*ph*, HaBacΔ*pif2*-Ac*pif2*-*ph*, HaBacΔ*pif3*-Ac*pif3*-*ph*, HaBacΔ*p74*-Sp*p74*-*ph*, HaBacΔ*pif1*-Sp*pif1*-*ph*, HaBacΔ*pif2*-Sp*pif2*-*ph* and HaBacΔ*pif3*-Sp*pif3*-*ph*.

**Table 1:** Primers for amplification of *pifs* from AcMNPV and SpltNPV genomes.

Primers	Sequences
Ac *p74* F	5’-cgggcatgcactctaaattcatgtattac-3’ (*Sph* I)
Ac *p74* R	5’-cgcggtaccagttccaagtaaatgaatc-3’ (*Kpn* I)
Ac *pif1* F	5’-ggggcatgcaatgttgatacgcattattcac-3’ (*Sph* I)
Ac *pif1* R	5’-cgcggtaccatgctcatgttgttatacagag-3’ (*Kpn* I)
Ac *pif2* F	5’-ggcgcatgcgtatagatagattgataacc-3’ (*Sph* I)
Ac pif2 R	5’-cgcggtaccgacttattgtttgtgttatc-3’ (*Kpn* I)
Ac pif3 F	5’-cggctcgagatcgccattgcaacacatcac-3’ (*Xho* I)
Ac pif3 R	5’-ggcggtacccagaatctcttacatttcagttg-3’ (*Kpn* I)
Splt *p74* F	5’-cggctcgaggataaataaatgaatatatgc-3’ (*Xho* I)
Splt *p74* R	5’-cgcggtaccctaatgaataataaacttttg-3’ (*Kpn* I)
Splt *pif1* F	5’-cggctcgagttcacaagagccatggtttag-3’ (*Xho* I)
Splt *pif1* R	5’-gcgggtaccgctaaatacaacgcttcgtcg-3’ (*Kpn* I)
Splt *pif2* F	5’-cgggcatgcaacagttgttcgcattgctg-3’ (*Sph* I)
Splt *pif2* R	5’-gcgggtaccacgaaaagtctaacaagtttatg-3’ (*Kpn* I)
Splt *pif3* F	5’-ccggcatgctcgtaatagattcatcgaacatg-3’ (*Sph* I)
Splt *pif3* R	5’-cgcggtacccaaacatcgaacagtgtccag-3’ (*Kpn* I)

Restriction sites are underlined.

### Transfection and infection

To produce the recombinant viruses, HzAM1 cells were seeded into tissue culture wells at a density of 5×10^5^ cells per well. Transfection was performed with 0.5 μg bacmid DNA using 10 μl lipofectin (Invitrogen). At 6 days post transfection (p. t.), the supernatant containing BVs was harvested by centrifugation to remove cell debris and used to infect a new batch of HzAM1 cells. The titer of each recombinant BV was determined by end point dilution assays (EPDAs).

### Electron microscopy (EM)

HzAM1 cells were infected with the individual *pif*-substituted recombinant HearNPVs or control virus vHaBac-*egfp-ph* respectively at an MOI of 5 TCID_50_ units/cell. Infected cells were processed for electron microscopy examination at 72 h p.i. as described previously [22].

### Western blot analyses of recombinant viruses

Polyclonal antibodies against HearNPV P74, PIF1, PIF2 and PIF3 were generated previously in rabbits [22]. To generate polyclonal antibodies against AcPIFs and SpPIFs, partial coding sequences of the *pifs* were amplified from AcMNPV or SpltNPV genomes. DNA fragments containing Ac*p74* (1–1251 nt), Ac*pif1* (124–1590 nt), Ac*pif2* (1–1146 nt), Ac*pif3* (85–612 nt), Sp*p74* (1–1287 nt), Sp *pif1* (109–1575 nt), Sp*pif2* (1–1146 nt), or Sp*pif3* (73–600 nt) were each cloned into the pET-28a expression vector. These truncated PIF proteins were expressed in *E*. *coli* BL21 cells and purified for immunizing rabbit to generate polyclonal antibodies.

HzAM1 cells were infected with vHaBac-*egfp*-*ph*, vHaBacΔ*pifs*-Ac*pifs*-*ph* or vHaBacΔ*pifs*-Sp*pifs*-*ph* at an MOI of 5. Infected cells were harvested at 96 hours post infection (h p.i.), separated on 12% SDS-PAGE and analyzed by Western blots. ODVs of AcMNPV and SpltNPV were used as positive controls. The PIFs-specific polyclonal antibodies were used as the primary antibodies and an alkaline phosphatase-conjugated goat anti-rabbit immunoglobulin (Gibco-BRL) was used as the secondary antibody. The final signal was detected by using a BCIP/NBT kit (Sino-America).

### Immunofluorescence assays (IFA)

HzAM1 cells were infected with vHaBac-*egfp*-*ph*, vHaBacΔ*pifs*-Ac*pifs*-*ph* or vHaBacΔ*pifs*-Sp*pifs*-*ph* at an MOI of 5. At 72 h p.i., cells were fixed with 4% paraformaldehyde and made permeable with 0.2% Triton X-100. After being blocked with 5% BSA, the cells were incubated with anti-PIFs polyclonal antibodies and then with Alexa Fluor^™^ 555-conjugated Goat Anti-Rabbit IgG H&L (Abcam) as the secondary antibody. Nuclei were stained with Hoechst stain. The subcellular localization of the PIFs was detected by fluorescence microscopy.

### Bioassays

Systemic infection was initiated by intrahaemocoelic injection of BVs into late third-instar *H*. *armigera* larvae as described previously [22]. About 10 μl of 10^6^ TICD_50_ units/ml of BVs was injected into the haemocoel of each larva. Grace’s medium was used as a negative control.

Oral infectivity of the recombinant viruses was detected by the droplet method with early third-instar *H*. *armigera* larvae described before [22]. The occlusion bodies (OBs) used for bioassay were harvested and purified from diseased larvae. In feeding assay, 10^8^ OBs/ml of each virus were used [34]. To determine median lethal concentration LC_50_ value, bioassays were conducted by exposing larvae to different virus concentrations: 1×10^3^, 3×10^3^, 1×10^4^, 3×10^4^, 1×10^5^, 3×10^5^, 1×10^6^, 3×10^6^, 1×10^7^, 3×10^7^, 1×10^8^, 3×10^8^ and 1×10^9^ OBs ml^-1^. Probit analysis was used to calculate LC_50_ values, 95% confidence limits and regression slopes. Data from two replicates were pooled to calculate the final LC_50_ values, as long as there was no significant difference between the LC_50_ values and regression slopes of the replicates. LC_50_ values between each pair of recombinant viruses were compared by the lethal dose ratio method of [35].

In all the above bioassay experiments, larvae were kept separately in 24-well plates and monitored daily until all larvae had either pupated or died as a result of virus infection. At least 48 larvae were used per treatment. All the bioassays were done in duplicates.

## Result

### Construction and characterization of pifs substituted HearNPVs

**Fig 1:**
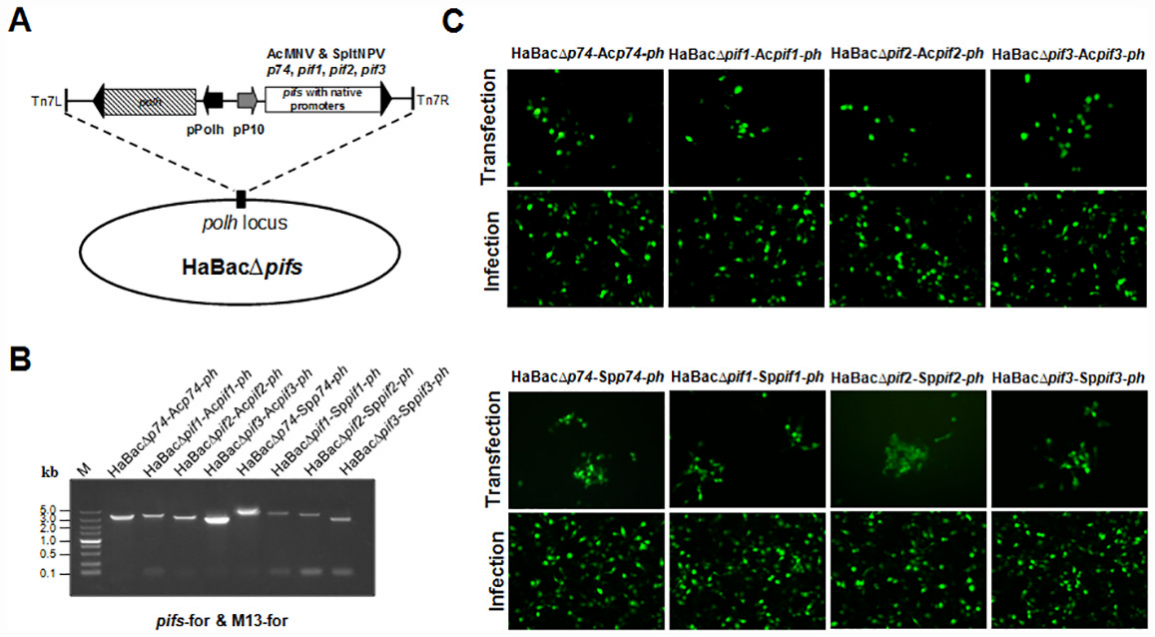
Construction and identification of HearNPV bacmids with substituted *pif*s and recombinant viruses. (A) Schematic overview of constructed HearNPV bacmids. (B) PCR identification of Ac*pif*s and Sp*pif*s genes in recombinant HearNPV bacmids. (C) HzAM1 cells were transfected with constructed HearNPV bacmids (upper panels) or infected with *pif*-substituted HearNPV recombinant viruses (below panels). EGFP was used to monitor the transfection and infection by fluorescence microscopy at 72 h p.t. and 72 h p.i.

**Fig 2:**
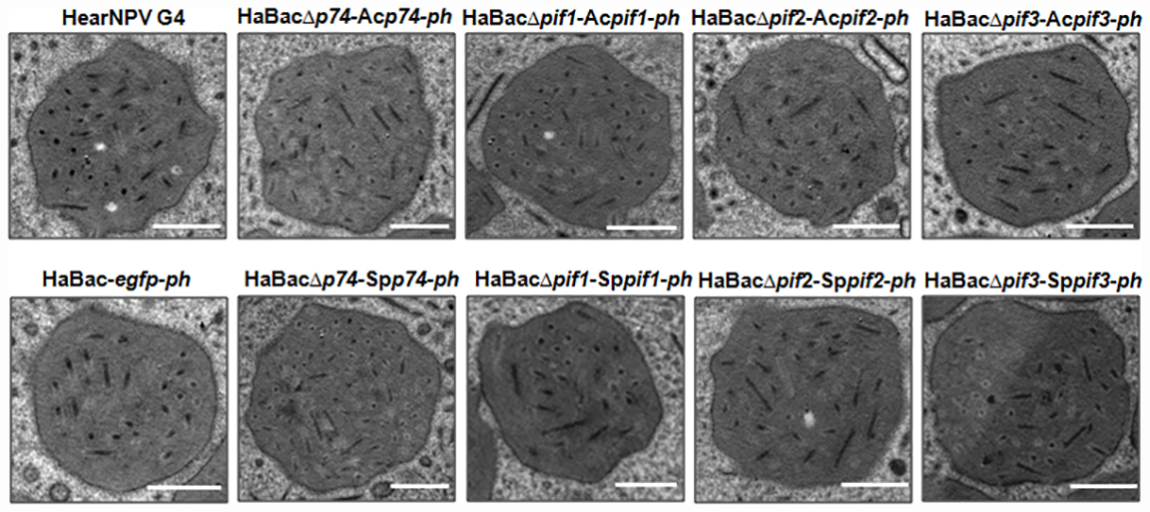
EM analysis of recombinant viruses-infected HzAM1 cells. HzAM1 cells were infected with the respective viruses at an MOI of 5. Cells were collected at 72 h p.i. and observed under a Hitachi H-7000 electron microscope operated at 75 kV. Bars, 500 nm.

In order to investigate the host specificities of baculoviral PIFs, the HearNPV bacmids, each with an individual *pif* deletion and containing *egfp* marker gene [22] were used as a backbone to insert the *pif* counterparts from AcMNPV and SpltNPV (Fig 1A). The recombinant bacmids HaBacΔ*pifs*-Ac*pifs-ph* and HaBacΔ*pifs-*Sp*pif-ph* were identified and verified by PCR (Fig 1B). To generate recombinant HearNPVs, each constructed bacmid DNA was used to transfect and then infect HzAM1 cells. The successful productions of infectious progenies from all the recombinant bacmids were characterized by the proliferation of green fluorescence (Fig 1C) and appearance of polyhedra (data not shown). Further EM observation showed that all the *pif*-substituted recombinant viruses formed normal OBs with embedded ODVs, much like the control virus (Fig 2).

### Expression of PIFs in infected HzAM1 cells

**Fig 3:**
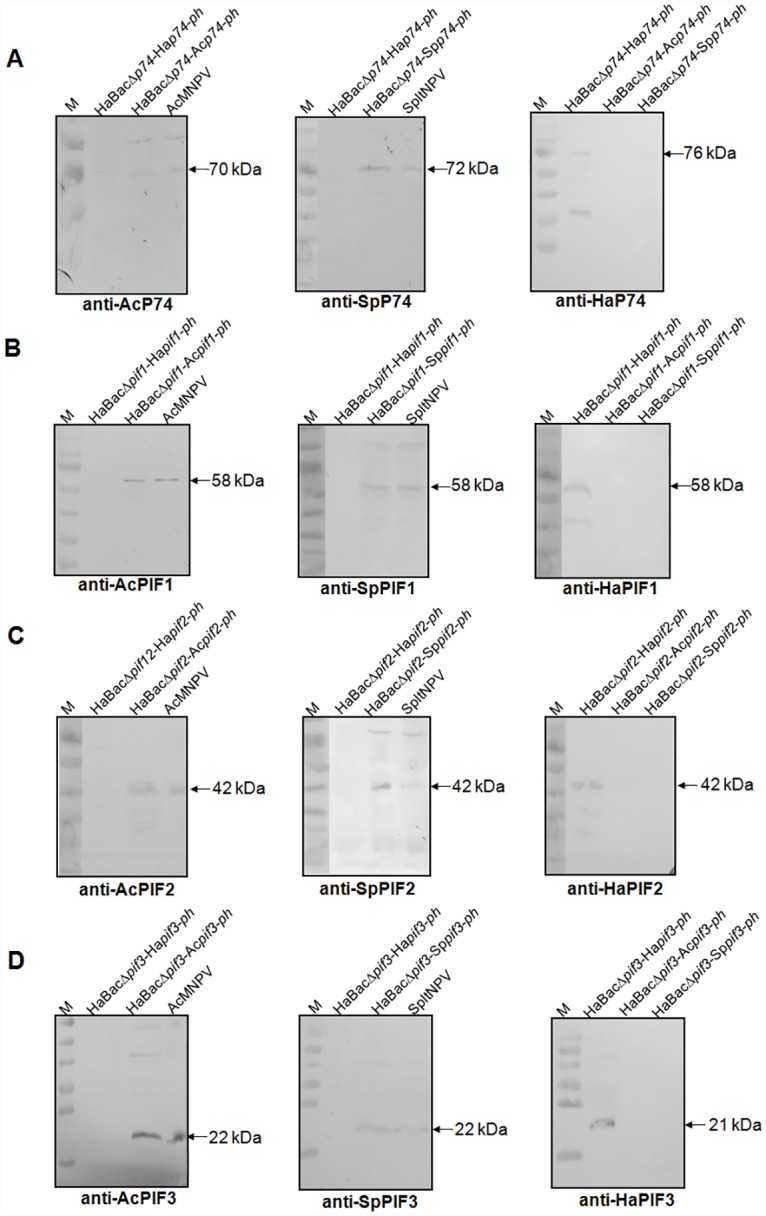
Expression of PIFs in infected cells. The different viruses were used to infect HzAM1 cells, and the cells were collected at 72 h p.i. for Western blots analysis using (A) anti-AcP74, SpP74 and HaP74 polyclonal antibodies; (B) Anti-AcPIF1, SpPIF1 and HaPIF1 polyclonal antibodies; (C) Anti-AcPIF2, SpPIF2 and HaPIF2 polyclonal antibodies; (D) Anti-AcPIF3, SpPIF3 and HaPIF3 polyclonal antibodies. HearNPV, AcMNPV and SpltNPV antibodies were indicated in the bottom of the blots and the molecular weight of PIFs are marked by arrows. M: Pre-stained protein molecular weight markers.

In cells infected with vHaBacΔ*p74*-Ac*p74*-*ph* or AcMNPV ODV, a band of the expected size of AcP74 (~70 kDa) reacted with anti-AcP74 antibody (Fig 3A-left). This band was absent in cells infected with vHaBacΔ*p74*-Ha*p74*-*ph* (Fig 3A-left). By using anti-SpP74 antibody, a band of the size of SpP74 (~72 kDa) was detected in extracts of vHaBacΔ*p74*-Sp*p74-ph* infected cells and SpltNPV ODVs, but not in vHaBacΔ*p74*-Ha*p74-ph* infected cells (Fig 3A-middle). Anti-HaP74 antibody detected a band of expected size of HaP74 (~76 kDa) in vHaBacΔ*p74-*Ha*p74*-*ph* infected cells, but not in those of vHaBacΔ*p74-*Ac*p74-ph* and vHaBacΔ*p74-*Sp*p74-ph* infected cells (Fig 3A-right). An additional band of ~40 kDa was detected in the vHaBacΔ*p74-*Ha*p74*-*ph* infected cells, which may represent a partially cleaved HaP74 (Fig 3A-right) (Huang *et al*., unpublished data).

Similarly, AcPIF1(~58 kDa), AcPIF2 (~42 kDa) and AcPIF3 (~22 kDa) as well as SpPIF1(~58 kDa), SpPIF2 (~42 kDa) and SpPIF3 (~22 kDa) were also expressed in cells infected with Ac*pif1*-, Ac*pif2-*, Ac*pif3-*, Sp*pif1-*, Sp*pif2-* and Sp*pif3-*substituted HearNPV recombinant viruses, but not in cells infected with vHaBacΔ*pif1*-Ha*pif1*-*ph*, vHaBacΔ*pif2*-Ha*pif2*-*ph* or vHaBacΔ*pif3*-Ha*pif3*-*ph* (Fig 3B–3D, left and middle panels). In contrast, HaPIF1 (~58 kDa), HaPIF2 (~42 kDa) and HaPIF3 (~21 kDa) were detected only in the cells infected with vHaBacΔ*pif1*-Ha*pif1*-*ph*, vHaBacΔ*pif2*-Ha*pif2*-*ph* or vHaBacΔ*pif3*-Ha*pif3*-*ph*, but not in samples of *pif*-substituted viruses (Fig 3B–3D, right panels). These results confirmed the correct expression of all the four heterologous SpPIFs and AcPIFs in the recombinant viruses-HzAM1 system.

### Localization of PIFs in infected HzAM1 cells

**Fig 4:**
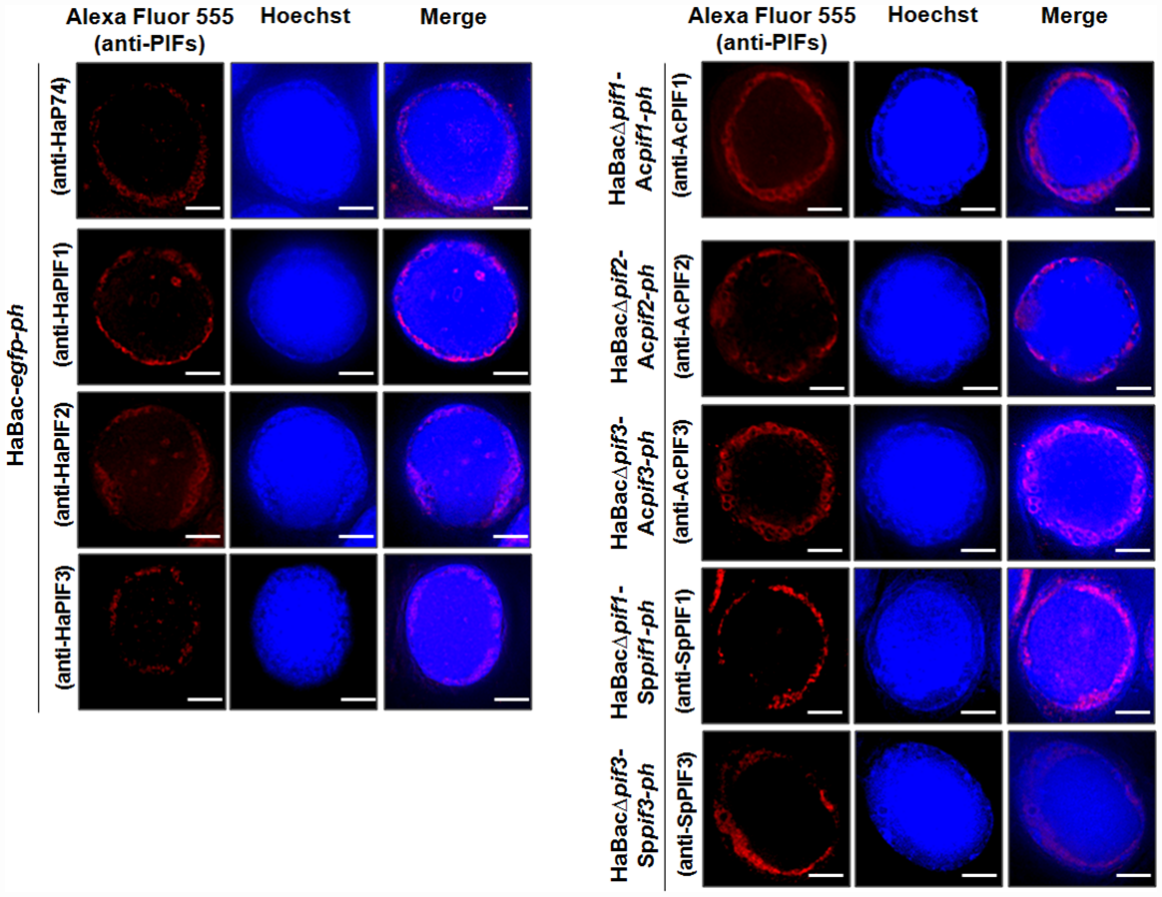
Subcellular localization of PIFs in infected cells. HzAM1 cells were infected with the parental control virus vHaBac-*egfp*-*ph* (left panel) or *pif*-substituted viruses (right panel). At 72 h p.i., cells were fixed, permeabilized and probed with the corresponding anti-PIF polyclonal antibody/Alexa Fluor^™^ 555-conjugated goat anti-rabbit antibody and viewed using a fluorescent microscope. Nuclei were stained with Hoechst stain. Bars, 5 μm.

IFA was performed to detect the subcellular localization of the heterologous PIFs in infected HzAM1 cells. As shown in Fig 4-left panel, the four PIFs of HearNPV were mainly localized to the nuclear ring zone region of their native host cells. Except for AcP74, SpP74 and SpPIF2, whose antibody was not suitable for use in IFA, the rest 5 heterologous PIFs, including AcPIF1, AcPIF2, AcPIF3, SpPIF1 and SpPIF3 also accumulated in the ring zone region of HzAM1 cells (Fig 4-right panel). Therefore, most of the tested Ac- and SpPIFs were properly transported to the nuclear ring zone region of HzAM1 cells.

### All viruses with substituted *pif*s, except vHaBacΔ*pif*3-Sp*pif*3-*ph* retained their systemic infectivity but lost their oral infectivity

To test the systemic infectivity of the *pif*-substituted viruses, supernatants containing BVs of parental controls, *pif*-substituted and *pif*-repaired viruses were injected into the haemolymph of late third-instar *H*. *armigera* larvae with a titer of 10^6^ TCID_50_ units/ml. All the recombinant viruses killed the tested larvae, suggesting that the *pif*-substituted viruses retained their systemic infectivity (data not shown).

To assess the oral infectivity of the *pif*-substituted viruses, preliminary feeding experiments were carried out. OBs (10^8^/ml) of the recombinant viruses isolated from infected larvae were fed to the third-instar *H*. *armigera* larvae by droplet method. The results showed that all the *pif*-substituted recombinants except vHaBacΔ*pif*3-Sp*pif*3-*ph* were not infective to *H*. *armigera* larvae even with such a high virus dose (Table 2).

**Table 2:** Feeding experiments of recombinant viruses in early third instar *H*. *armigera* larvae.

Virus	Test 1 Dead/Tested	Test 2 Dead/Tested
HaBac-*egfp*-*ph*	48/48	48/48
HaBac*p74-*Ac*p74*-*ph*	0/48	1*/48
HaBac*pif1-*Ac*pif1*-*ph*	2*/48	0/48
HaBac*pif2-*Ac*pif2*-*ph*	0/48	0/48
HaBac*pif3-*Ac*pif3*-*ph*	3*/48	2*/48
HaBac*p74-*Sp*p74*-*ph*	0/48	1*/48
HaBac*pif1-*Sp*pif1*-*ph*	2*/48	0/48
HaBac*pif2-*Sp*pif2*-*ph*	0/48	0/48
HaBac*pif3-*Sp*pif3*-*ph*	46+1*/48	47/48
Mock	0/48	0/48

*The death was not due to virus infection.

OB concentration = 1 × 10^8^ OBs/ml.

**Fig 5:**
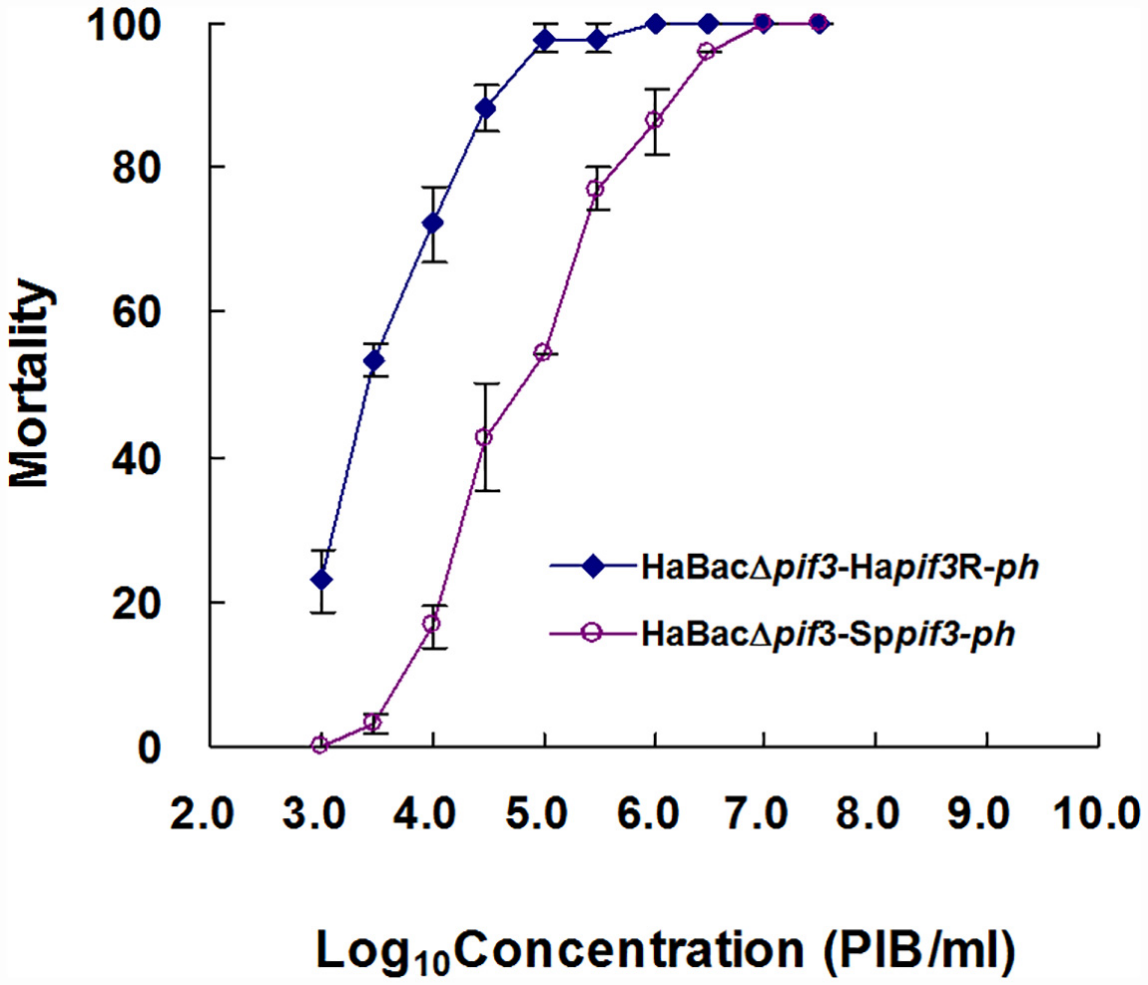
Bioassays. Forty-eight third-instar *H*. *armigera* larvae were infected with different concentrations of each recombinant virus by the droplet method. The final mortality for each virus concentration was calculated. Each data point represents the mean value from three separate infections; error bars indicate standard deviation.

A bioassay experiment was further performed to determine the LC_50_ value. As shown in Fig 5 and Table 3, the LC_50_ values of vHaBacΔ*pif*3-Sp*pif*3-*ph* was 7.38×10^4^ OBs/ml, which is about 23-fold higher than that of the control virus vHaBacΔ*pif*3-Ha*pif*3-*ph* (LC_50_ = 3.23×10^3^ OBs/ml). Statistical analyses indicated that the LC_50_ values of HaBacΔ*pif*3-Sp*pif*3-*ph* was significantly different from that of vHaBacΔ*pif*3-Ha*pif*3-*ph* (*P* < 0.05). The data reveal that SpltNPV PIF3 only partially substituted the function of HearNPV PIF3. Altogether, these results suggested that, although the *pif*s are conserved in baculoviruses, substitution of individual HearNPV *pifs* with homologues from AcMNPV or SpltNPV did not yield a virus with efficient oral infectivity, indicating the role of PIF proteins in host specificity.

**Table 3:** LC_50_ and regression slopes of concentrations-mortality of recombinant viruses.

Virus	LC_50_ (PIB/ml)	95% Confidence limit (PIB/ml)	Regression Slope
HaBac*pif3* -Ha*pif3*R-*ph*	3.23×10^3^	(2.11–4.60)×10^3^	1.197
HaBac*pif*3-Sp*pif3-ph*	7.38×10^4^ *	(5.32–1.02)×10^3^	1.158

*represent the significant difference between the LC_**50**_ values of these two viruses compared by the lethal dose ration method [35].

## Discussion

We studied the host specificity of HearNPV *p74*, *pif1*, *pif2* and *pif3* by substituting with their counterparts from AcMNPV or SpltNPV. Transfection-infection experiments showed that all the recombinant viruses produced BVs infective to cell culture. Intrahaemocoelic injection of BVs into *H*. *armigera* larvae confirmed that all the recombinant viruses retained their systemic infectivity. However, bioassay results demonstrated that only vHaBacΔ*pif*3-Sp*pif*3-*ph* could infect *H*. *armigera* larvae via oral route but with significantly decreased virulence. All the other recombinant viruses with substituted *pif*s completely lost their oral infectivity.

In fact apart from AcMNPV and SpltNPV, the *p74*, *pif1*, *pif2* or *pif3* gene of two other group II alphabaculovirus, *Spodoptera exigue* (Se)MNPV and *Chrysodeixis chalcites* (Chch)NPV, were also used to substitute their counterparts in HearNPV. Similar experiments were carried out, and none of the above Se*pifs* or Chch*pifs* rescued the oral infectivity of *pifs*-deleted HearNPV (data not shown). These data suggest that the host specificity of P74, PIF1, PIF2 and PIF3 may be universal in baculovirus. It is important to mention the insect also plays an important role in host specificity. Only those with receptors in their midgut columnar epithelial cells specific to certain PIFs allow that virus to gain access to these cells. In other words, host specificity is a mutually dependent property of the virus and the host.

**Fig 6:**
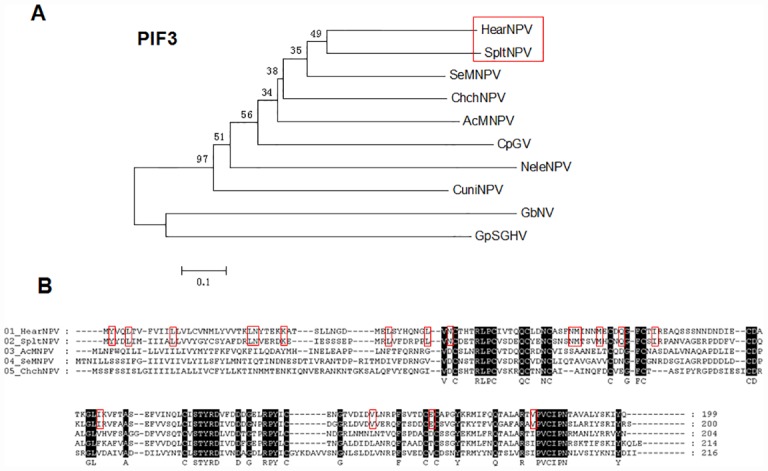
Phylogeny and the alignment of the related PIF3 proteins. (A) Neighbour Joining tree of PIF3s of baculoviruses, nudivirus and SGHV. Bootstrap value: 1000. (B) Sequence alignment of PIF3s of HearNPV, SpltNPV AcMNPV, SeMNPV and ChchNPV. The conserved amino acids of HearNPV and SpltNPV PIF3 are shown in red columns. GenBank accession numbers for the PIF3 proteins: NP_075167.1 (HearNPV), NP_258375.1 (SpltNPV), NP_037810.1 (SeMNPV), NP_054145.1 (AcMNPV), YP_249714.1 (ChchNPV), NP_148819.1 (CpGV), YP_025266.1 (NeleNPV), NP_054145.1 (CuniNPV), YP_001111270.1 (GbNV), YP_001687024.1 (GpSGHV).

As PIF3 of SpltNPV seems to be an exception since it can partially rescue the oral infectivity of HearNPV, we further studied the phylogeny of PIF3. As shown in Fig 6A, SpPIF3 is evolutionarily closer to HaPIF3 compared to the other examined PIF3 proteins, which is in accordance with the result of other core genes trees [36, 37]. Sequence alignment also showed that the amino acid (aa) identity of PIF3 N-terminus between SpltNPV and HearNPV is relatively higher than with other baculoviruses (Fig 6B). This may provide a possible explanation for the result of partial function rescue of HaPIF3 by SpPIF3. Among PIF74, PIF1, PIF2 and PIF3, the latter shows the lowest conservation. Sequence analyses show that the aa identity among different baculoviruses is 37–63% for P74, 31–59% for PIF1, 45–71% for PIF2 and 31–58% for PIF3 (data not shown), indicating that PIF3 is under less stringent selection pressure than that of the other PIFs. Unlike P74, PIF1 and PIF2, which are believed to be involved in the virus binding to a specific receptor on the midgut cells, PIF3 is suggested to mediate necessary interaction between the host and virus downstream of binding process [12]. The different function of PIF3 may also contributed to its less stringent host specificity in comparison to that of P74, PIF1 and PIF2, at least in the case of SpPIF3.

P74, PIF1, PIF2 and PIF3 constitute the core components of the PIF complex [24, 25]. Yeast two hybrid experiments also showed that some PIF proteins interact with other PIFs [38]. Therefore, the function of an individual PIF may be dependent on proper recognition and interaction with other PIFs. And this may explain why substituting an individual PIF does not mediate oral infection. It will be interesting to test possible rescue of oral infectivity by substituting a whole set of PIFs instead of just individual PIFs. It also remains to be studied whether the PIF4, PIF5 and PIF6 also exhibit similar host specificity.

In summary, by constructing a series of *pif*s-substituted pseudotyped baculoviruses, we for first time characterized that PIF proteins (P74, PIF1, PIF2 and PIF3) of baculoviruses have strict host specificities. Only SpltNPV PIF3 is able to partially substitute for the function of HearNPV PIF3. The data provided evidence that PIFs are crucial for host range of baculoviruses. Further research will focus on elucidation of the interaction networks among PIFs and the midgut host factors during oral infection. We are presently investigating a system that will allow substituting several PIFs simultaneously to gain further insight into host specificity. These attempts will help disclose the oral infection mechanism and the host specificity of baculoviruses.
